# Rehabilitation facilities' research needs and knowledge transfer in Southwest Germany: Data of the REHA-KNOWS study

**DOI:** 10.1016/j.dib.2023.109632

**Published:** 2023-09-27

**Authors:** Urs A. Fichtner, Nicole Wimmesberger, Matthias Sehlbrede, Erik Farin-Glattacker

**Affiliations:** Institute of Medical Biometry and Statistics, Section of Health Care Research and Rehabilitation Research, Faculty of Medicine and Medical Center – University of Freiburg, Hugstetter Straße 49, 79106 Freiburg im Breisgau, Germany

**Keywords:** Knowledge-to-practice gap, Participatory research, Determinants, Evidence-based medicine, Knowledge transfer, Attitudes, Perceptions, Health services research

## Abstract

The REHA-KNOWS study was conducted to identify research needs, to explore attitudes and barriers towards health services research as well as to investigate knowledge transfer strategies in rehabilitation facilities in Southwest Germany. We designed a short online survey with 18 questions. From March to May 2023, we surveyed representatives of rehabilitation facilities in two German Federal States (Baden-Wuerttemberg and Saarland) using an online questionnaire provided via Unipark^Ⓡ^. The dataset contains all responses from n=88 individuals. We applied a list-based sampling approach and contacted n=206 rehabilitation facilities in total. Data collection started on March 2^nd^ and the last response was received on May 2^nd^. As this sampling strategy allows multiple answers per facility, we applied an anonymized coding system to identify the affiliation of each respondent. In total, the 88 responses come from 74 centers. The dataset includes information on characteristics of the facility where the respondents work, the perceived benefits and barriers regarding health services research in practice, the need for research on specific topics and the transfer strategies established within the facilities. Analyses of these topics were performed in a descriptive and exploratory manner. This data offers the potential to be linked with data resulting from future research in this field in other Federal States of Germany. Further subgroup analyses can be performed with this dataset for specific research questions.

Specifications Table

**Table:** 

Subject	Social Sciences - Health
Specific subject area	Cross-sectional online survey among representatives of rehabilitation facilities
Data format	Raw Processed (for data publication)
Type of data	Table
Data collection	Data was acquired through a self-administered online survey provided via Unipark^Ⓡ^. Respondents were approached via invitation e-mail based on a self-produced list representing the basic population of rehabilitation facilities (n=206) in two German Federal States (Baden-Wuerttemberg and Saarland). The dataset and the questionnaire are published in ZENODO and can be accessed without restriction here: https://doi.org/10.5281/zenodo.8147221[1] The questionnaire contained self-developed instruments which were discussed in advance with 4 professionals having expertise in the fields of psychology, health care research, sociology and public health. The questionnaire was provided in German only, an English translation can be found in the repository. Respondents were asked to fill out the questionnaire within 20 days, a reminder was sent after 10 days. Since the sampling strategy addressed different representatives of each facility, multiple answers per center were possible. However, any respondent was able to participate only once.
Data source location	· Institution: University of Freiburg, Medical Faculty · City/Town/Region: Freiburg im Breisgau, Baden-Wuerttemberg · Country: Germany Respondents are associated with rehabilitation facilities located in Baden-Wuerttemberg and Saarland: NUTS-1-Codes (2021) DE1 and DEC *(*Hintergrund - NUTS - Systematik der Gebietseinheiten für die Statistik - Eurostat (europa.eu))
Data accessibility	Repository name: ZENODO Data identification number: 8147221 Direct URL to data: https://doi.org/10.5281/zenodo.8147221[1]

## 1 Value of the Data

• Data can be linked to subsequent surveys in this field covering other German Federal States.
• Data can be used by other researchers for subgroup analyses.
• This study was used for a first exploration of the distribution parameters and the facture structure of a newly developed instrument to measure attitudes towards the role of health services research in the practice of rehabilitation facilities. The implemented questionnaire can be used by other researchers for further development and testing in other studies.

## 2 Data Description

In total, the dataset includes responses from n=88 individuals representing n=74 rehabilitation facilities. Thus, the coverage rate is 36% of the initially contacted facilities in Baden-Wuerttemberg and Saarland.

The dataset comprises 78 variables that can be grouped into several sections (see Table 1):

**Table 1:** Overview on topics covered in the dataset.

Study-related / administrative variables	facilityid, i_ds, datetime date_of_last_access,
Respondent / facility characteristics	i_01, i_01a, i_02, i_03_x, Other_Diseases, i_04, i_05, size
Experience with health services research	i_08, i_09, i_10
Attitudes towards health services research	i_11_x
Research topics of interest	i_12_x, i_13
Transfer strategies	i_14_x, i_15, i_16

The questionnaire is published together with the dataset in English.

## 3 Experimental Design, Materials and Methods

For this study, we designed a short online survey of 18 questions. These were all self-developed and consented within a team of experts in sociology, psychology, public health, empirical social sciences and health services research. We applied a list-based sampling approach and contacted n=206 rehabilitation facilities in total. The sampling list was generated through internet search using search engines and online databases (https://www.qualitaetskliniken.de; https://www.rehakliniken.de). We generated a list with e-mail addresses from each facility trying to identify 3 representatives for each unit: a) from the medical professional staff, b) from the administrative, management level, c) a generic contact e-mail address. Where possible, we applied common e-mail composition logic following the guidance we found on the websites of the facilities (e.g. “Firstname.Lastname@...”) to complete missing data. In the selection of e-mail addresses, we prioritized a) and b) since they were associated with a specific person within the facility. We randomized them for the first sampling approach, so that 50% of the representatives contacted first were medical professionals and 50% were from the administrative management level. If we did not receive an e-mail requesting no further contact or if the list contained incorrect or missing information, we contacted the facilities three times in total with one reminder per e-mail address after 10 days. In total, we sent out 457 e-mails (wave 1: 206, wave 2: 160, wave 3: 91). 73.7% of all respondents did not open the link included in the e-mail invitation. 7% opened the link and started the survey, but stopped. Furthermore, we recorded whether respondents participated before or after the reminder e-mail: 58% of our sample responded after the initial invitation, 42% participated after the reminder. Data collection started on March 2^nd^ and the last response received was from May 2^nd^. As this sampling strategy allows for multiple responses per facility, we applied an anonymized coding system to identify the affiliation of each respondent. In total, the 88 responses originated from 74 centers.

## Limitations

We explicitly addressed representatives of rehabilitation facilities, who have positions with personnel responsibility. Thus, our data might not represent all the perspectives from practice in case that opinions might differ from those in other occupational position.

## Ethics Statement

Data collection was carried out in accordance with the Declaration of Helsinki. Participation in the survey was only possible after digital informed consent to our privacy policy. Where necessary, we deleted variables that could lead to the identification rehabilitation facilities, but as these were administrative variables, this did not affect the content of the dataset. Due to the anonymous data collection mode, ethical approval was not required for this study.

## CRediT authorship contribution statement

**Urs A. Fichtner:** Conceptualization, Methodology, Software, Data curation, Writing – original draft, Visualization, Investigation, Validation. **Nicole Wimmesberger:** Conceptualization, Methodology, Software, Data curation, Writing – original draft, Visualization, Investigation, Validation. **Matthias Sehlbrede:** Writing – review & editing, Methodology, Software. **Erik Farin-Glattacker:** Supervision, Writing – review & editing.

## Data Availability

The REHA-KNOWS Dataset (Original data) (ZENODO).
